# A Stable Dual-wavelength Thulium-doped Fiber Laser at 1.9 μm Using Photonic Crystal Fiber

**DOI:** 10.1038/srep14537

**Published:** 2015-10-12

**Authors:** M. R. K. Soltanian, H. Ahmad, A. Khodaie, I. S. Amiri, M. F. Ismail, S. W. Harun

**Affiliations:** 1 Photonics Research Centre, University of Malaya, 50603 Kuala Lumpur, Malaysia

## Abstract

A stable dual-wavelength thulium-doped fiber laser operating at 1.9 μm using a short length of photonic crystal fiber (PCF) has been proposed and demonstrated. The photonics crystal fiber was 10 cm in length and effectively acted as a Mach-Zehnder interferometry element with a free spectral range of 0.2 nm. This dual-wavelength thulium-doped fiber laser operated steadily at room temperature with a 45 dB optical signal-to-noise-ratio.

Thulium-doped fiber lasers (TDFLs) have received increasing attention within the field of laser research in recent years due to their applicability in areas such as gas detection^1^, mechanical processing of polymers^2^, optical communication^3^, light detection and ranging (LIDAR), and remote sensing. One particular cause for this interest lies in the ability of TDFLs to emit a laser beam that poses no damage to eyes, since the output wavelength region spans 1.9 to 2.1 μm. This feature provides TDFLs with a significant advantage for use in applications that involve light propagation in a free space or in the atmosphere, such as free space optical communication and LIDAR atmospheric water vapor profile measurement^1^.

Many researchers have investigated various TDFL configurations to determine suitability for particular types of applications. One configuration where dual-wavelength TDFLs operate at 2 μm is considered as an attractive proposition for dual-wavelength laser generation, since most previously reported dual wavelength fiber lasers were operating at the 1.0 μm and 1.55 μm bands. Research efforts associated with this configuration have been additionally driven by an expected broad applicability that includes ranging systems^4^, communication, microwave photonics^5^,^6^ and terahertz generation^7^. Moreover, progress on all-fiber dual wavelength generation from TDFLs is increasingly feasible as 2 μm compatible fiber components become readily available.

Similarly to the case of ytterbium-doped fiber lasers and erbium-doped fiber lasers, which operate at 1.0 μm and 1.55 μm respectively, achieving dual wavelength generation in the 2.0 μm wavelength region with TDFLs remains challenging due to strong mode competition caused by homogeneous gain broadening in the gain media. Various approaches for overcoming this setback have been reported for erbium-doped fiber lasers, with examples including four wave mixing^8^, polarization hole burning^9^ and cascaded stimulated Brillouin scattering^10^, although approaches for the comparable case of TDFLs have received scant attention.

Zhou *et al.* have proposed a stable dual-wavelength TDFL based on cascaded fiber Bragg gratings^11^, whereby the laser wavelengths are determined by the fiber Bragg gratings (FBG) and thus the wavelength spacing is fixed by the central wavelength of the FBGs pair. On the other hand, a dual-wavelength laser proposed in^12^ that employs a Sagnac loop mirror made by a 145.5 cm polarization maintaining fiber is switchable, tunable, and operates stably at room temperature. Another configuration of tunable dual-wavelength TDFL is demonstrated by^13^, wherein a high birefringence FBG stabilizes the dual-wavelength lasing produced when the polarization state of each wavelength is altered via a polarization controller.

A large number of researchers have investigated the distinctive properties of photonic crystal fibers, such as wide range single mode operation, dispersion flexibility, large mode area and its application in multi-wavelength generation^14^,^15^,^16^,^17^. In most cases, those properties were proven to be wavelength dependent and equivalent to the behavior of wavelength-selective filters^18^,^19^.

In this paper, a stable dual-wavelength TDFL operating at 1.9 μm is proposed and demonstrated with sets of stable DWFL output obtained via a short length photonic crystal fiber (PCF) incorporated in a ring cavity setup. The use of PCF brings advantages of flexibility and wavelength-dependent characteristics, thus making the material an almost ideal choice as a wavelength selective filter for TDFL. This work is synchronous with previous efforts to generate microwave signals from fiber optic elements for use in radio over fiber (ROF) applications, which are mentioned in^16^,^17^,^18^ for the 1.5 μm region. The particular interest in the development of the 1.9 μm source is in anticipation of spectroscopic analysis applications of various gases, in particular for high sensitivity trace gas sensing of CO_2_, N_2_O, H_2_CO, and HBr^20^.

## Laser Configuration

**Figure 1:**
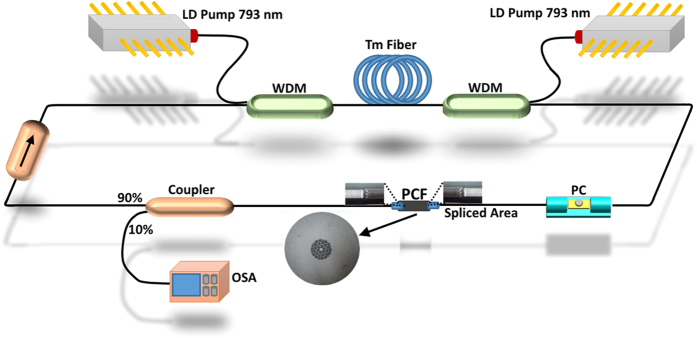
Configuration of the dual-wavelength TDFL at 2 μm with photonic crystal fiber based on the Mach Zehnder interferometry effect (drawn by coauthors, M. R. K. Soltanian and I. S. Amiri).

The configuration of the proposed dual-wavelength fiber laser based on polarization-dependent loss control is shown in Fig. 1. Components of this laser included a 793/2000 nm wavelength division multiplexer (WDM) that had an attached discrete 793 nm laser diode pump, followed by 2 meters of thulium-doped fiber (TDF), and subsequently a repeated arrangement of WDM and attached pump, a polarization controller, a 10 cm length of photonic crystal fiber, a 10:90 fiber based coupler, and an isolator that closed the fiber laser ring. The 793 nm pump outputs were coupled into the laser cavity by the WDMs in a bidirectional pumping configuration, while the TDF was pumped bi-directionally in the core via the two 793 nm laser diodes.

The combination of both laser diodes allowed for approximately 350 mW of pump power. The gain medium used in this setup was a 2 meter single mode TDF manufactured by Nufern Corporation, and had an absorption coefficient of 27 dB/m at 793 nm, core diameter of 9 μm and cutoff wavelength of 1750 nm. In order to obtain a more stable lasing condition, the laser was forced to operate unidirectionally via an isolator. The polarization controller provided a capability to switch the lasing wavelengths’ polarization state from one to another, which triggered a polarization dependent loss in the setup and consequent excitation of the dual-wavelength laser. As shown by the micrograph in Fig. 1 (inset), the PCF cross-section structure consisted of a solid core with a diameter of 4.37 μm and a surrounding ring of air holes, each having 5.06 μm diameter and a separation of 5.52 μm between the holes. The internal structure of the PCF was responsible for the separation of incoming light into several modes that subsequently propagated in different paths. The combination of these modes at the other end of the PCF allowed the PCF to perform as a Mach-Zehnder interferometer (MZI). In order to measure and analyze the laser output, a 10:90 fused fiber coupler was used to tap 10% of the lasing power from the cavity while feeding back the remaining 90% to the cavity.

## Results and Discussion

**Figure 2:**
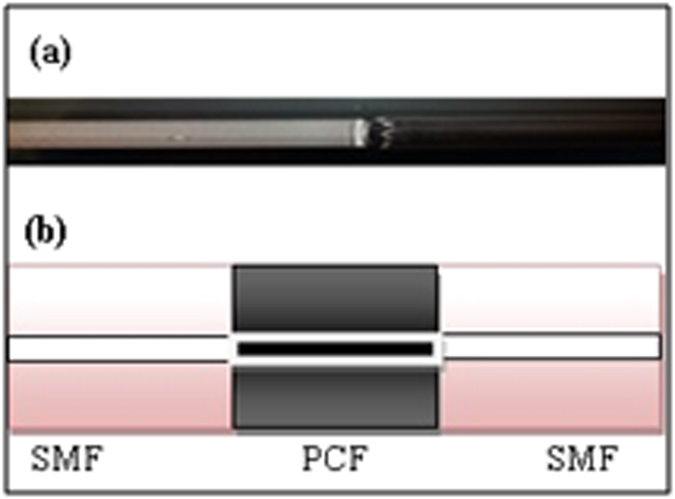
(**a**) Image from the collapsed region after splicing. (**b**) Schematic diagram of the MZI filter.

A compact MZI was constructed by fusion splicing a 10 cm long PCF between two segments of single mode fiber as shown in Fig. 2(a). The schematic diagram depicted in Fig. 2(b) illustrates the internal structure of the interferometer. Splicing the PCF and single mode fiber manually via a Fujikura 45 PM splicer caused a collapse of the air holes of the PCF. The two spliced points were located in series, and the length of the PCF determined fringe spacing. The splice loss was measured using a Thorlabs tunable laser kit (model TLK-L1900M) as a tunable laser source (TLS) that operated from 1870 nm to 1930 nm. The output laser was set at 1900 nm and a Thorlabs power meter (model PM100) was used to measure the output power during splicing performed by a Fujikura 45 PM splicer. The minimum splicing loss achieved was 1 dB for each splicing area.

**Figure 3:**
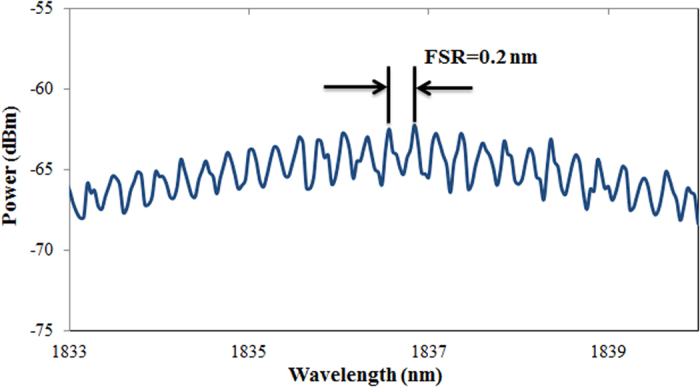
Transmission spectrum of the MZI.

The resulting transmission spectrum of the MZI, which is shown in Fig. 3, allowed for measurement of the free spectral range (FSR) as 0.2 nm.

**Figure 4:**
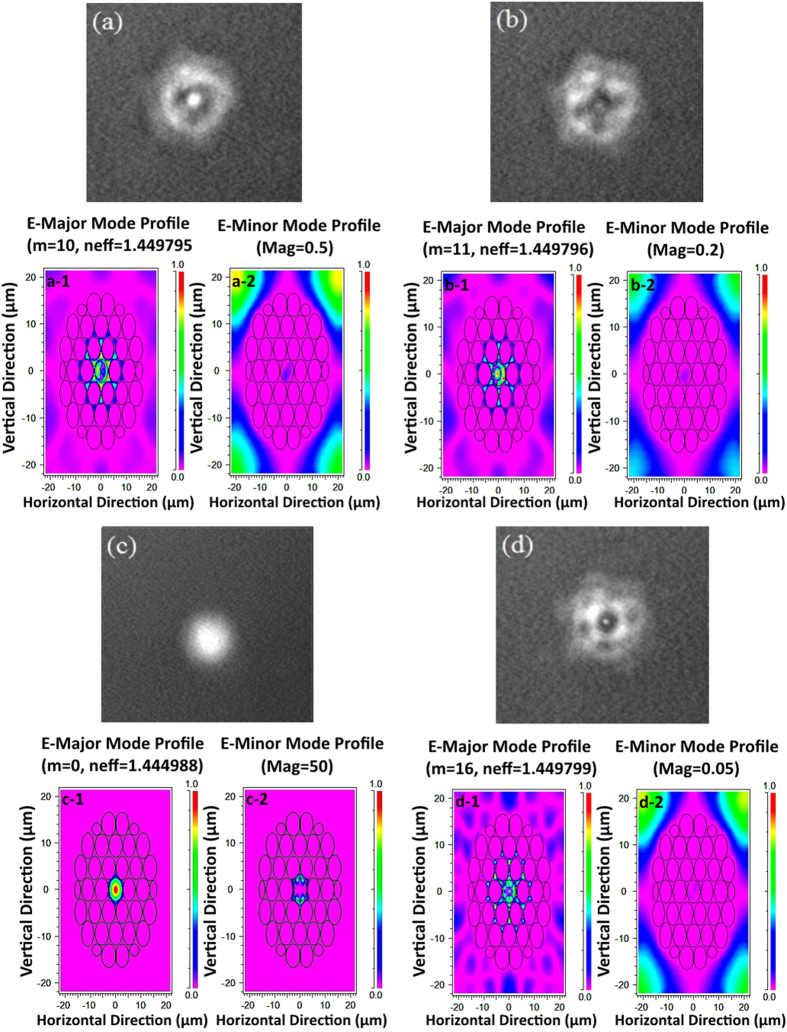
(**a–d**) Image captured by Electrophysics Micron Viewer Model 7290A for four different polarization states variations via a PC while a SLM laser source launched in the wavelength of 1850 nm. (a-1), (a-2), (b-1), (b-2), (c-1), (c-2), (d-1) and (d-2) are the modelling results field profiles for the fundamental quasi-TM modes corresponding to the experimental results for the modes m = 10, 11, 0 and 16 respectively.

The first collapsed region served to diffract the traversing fundamental mode, which resulted in the core and cladding modes becoming excited in the few modes PCF section as shown in Fig. 4(a–d). These four different polarization states images were captured via an Electrophysics Micron Viewer Model 7290A while a single longitudinal mode (SLM) laser lased at a wavelength of 1850 nm. Fig. 4(a-1), (a-2), (b-1), (b-2), (c-1), (c-2), (d-1) and (d-2) show the modeling results of the field profiles for the fundamental quasi-TM modes calculated with the vector beam propagation method (BPM). The modeled results are completely in agreement with the experimental results for the modes m = 10, 11, 0 and 16 respectively, and these agreed results show that the fundamental mode undergoes diffraction and becomes exited in a few modes. A portion of the fundamental core mode of PCF subsequently became coupled to a single mode or several cladding modes of the PCF. A variety of optical paths that corresponded to the different arms of MZI emerged due to the effective refractive index of the cladding being smaller than the core. The fundamental and cladding modes possessed dissimilar phase velocities and hence accumulated a phase difference along the PCF, whereby the magnitude of this phase difference was dependent on the length of PCF and the wavelength of the guided light.

The phase shifting that occurs within a physical length is equal to the product of different effective refractive indices of core and cladding modes within the length. When the propagating modes reached the subsequent collapsed area of the PCF, the cladding modes became coupled back to the core mode. Since the phase difference and the phase velocities are wavelength dependent, the optical power transmitted by the interferometer is at a minimum at certain wavelengths and maximum at others. The separation between consecutive peaks of a two-mode interferometer is given by 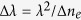, where Δ*n*_e_ is the effective refractive indices difference between the core and cladding modes. The core and the cladding of the PCF played the role of arms within an MZI, while the collapsed points acted as couplers splitting or combining light powers at the input and output of the interferometer.

**Figure 5:**
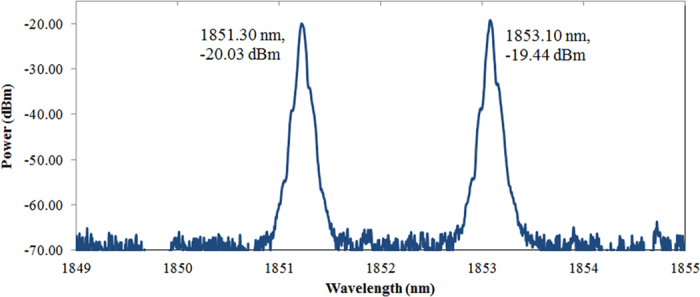
Optical spectrum of dual-wavelengh fiber laser at wavelengths of 1851.30 nm and 1853.10 nm with peak powers of −20.03 dBm and −19.44 dBm respectively.

Fine-tuning the polarization controller described in section 2 allowed for a dual-wavelength laser with nearly identical peak powers of −20.03 dBm and −19.44 dBm at 1851.30 nm and 1853.10 nm respectively, as measured from the 10% port and shown in Fig. 5. Insertion of a polarization controller into the cavity resulted in wavelength-dependent polarization rotation and a consequent diversification of polarization states across multiple wavelengths in the TDFL. If no changes occurred in the polarization states, all different wavelengths would remain in approximately identical polarization states in the TDF.

Increasing the number of peaks oscillating in the cavity necessitated the use of a polarization hole burning (PHB) technique^21^. Light entering the TDFL did not have the same polarization state, and this resulted in greatly enhanced PHB and a large reduction in the homogeneous linewidth of the TDF. An ensuing suitable adjustment of the polarization controller led to stable dual-wavelength lasing states at room temperature.

The distribution of the modes in the PCF is totally dependent on the polarization states of the modes entering the collapsed region created by splicing with SMF fiber. Each mode will be divided into several modes within the core or the cladding of the PCF. The in-fiber PCF-MZI is an in-line, all-fiber, and coupler-free device. In the role of a delay interferometer (DI), this in-fiber PCF has a relative delay governed by the index difference between its core mode and cladding mode. The index difference between the core mode and the cladding mode can be quite large (usually larger than 0.01), which is due to the unique air-hole structure of the PCF, and implies that a short PCF can be used to introduce a large delay while maintaining optical attenuation of the cladding mode at a relatively low level^22^. Hence, the two higher-order modes, whether in the cladding or core, have significantly different propagation constants, which results in these modes having highly dissimilar travel speeds along the PCF and interference between each other in the subsequent collapsed area of the PCF.

**Figure 6:**
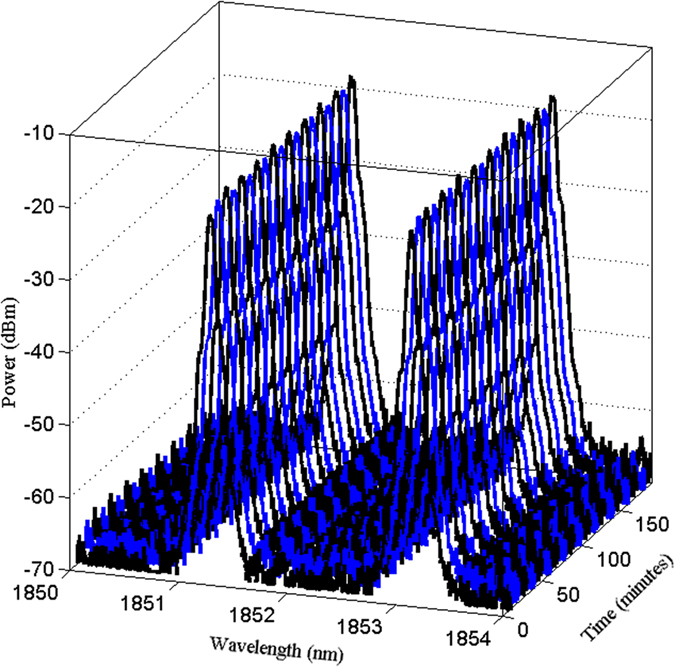
Stability of dual-wavelength fiber laser with spacing of 1.8 nm at 1851.30 nm and 1853.10 nm over 180 min with interval scanning of every 10 min.

The simple design of the dual wavelength fiber laser allowed for increased stability of the setup via the short ring cavity of about 6 m, along with high repeatability due to the inclusion of a PC. As can be observed, the wavelength spacing illustrated in Fig. 3 is dissimilar to that in Fig. 5, and this difference is attributed to the adjustment of the PC, which will either increase or decrease the spacing between the two wavelengths. In this manner, the polarization states of the modes play a key role to generate dual-wavelength fiber laser. Each (l, m, q) mode has two degrees of freedom, corresponding to two independent orthogonal polarizations. These two polarizations are regarded as two independent modes. The two polarization modes of the same l and m have the same spatial distributions. If the resonator and the active medium provide equal gains and losses for both polarizations, regardless of wavelength spacing, the laser will oscillate on the two modes simultaneously, independently, and with the same intensity^23^. Figure 6 illustrates the stability of the generated dual-wavelength over 180 minutes with an interval scanning of every 10 minutes in order to closely monitor the generated dual-wavelength lasing. It can be observed from this figure that the optical signal-to-noise ratio for both peaks is about 45 dB.

**Figure 7:**
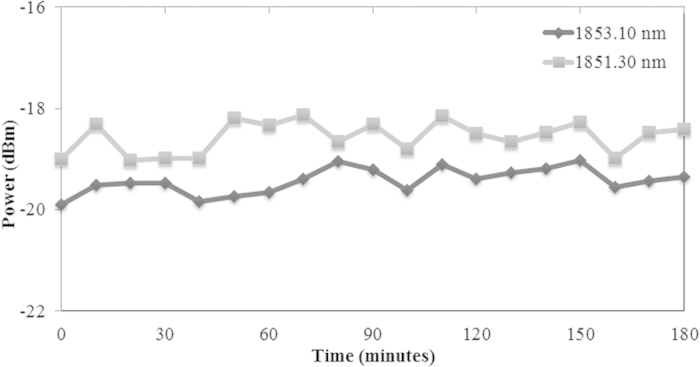
Optical power fluctuation for both lasing wavelengths at 1851.30 nm and 1853.10 nm over 180 minutes.

The power stability of this dual-wavelength was measured and the results shown in Fig. 7. As shown in Fig. 7, the power of the dual-wavelength fiber laser is very stable and the fluctuation is in the range of 1 dB. In general there are several solutions to increase the output power: ensure the cavity is at optimum length, exploit the inherent high absorption characteristics of an incorporated TDF, increase the pump power, reduce the total loss in the setup e.g. by minimizing splicing loss, and extract a higher portion from the ring laser cavity.

The most appropriate means for increased power is determined by the specific circumstances. For instance, if the output power versus the pump power curve is linear in the highest power region, then increasing the pump power is the simplest way to increase the output power linearly. If the curve is nonlinear, such as the slope reducing as the pump power increases, then the pump output is not fully absorbed and so we should increase the length of the active doped fiber. If there is a limitation to increasing the pump power, then efforts should be made to improve the laser efficiency and/or the lasing threshold, since improvements to both these characteristics occur when cavity losses are reduced. The laser output power equation is^24^:


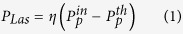


where 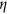 is the slope efficiency, 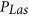 is laser output power, 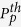 is pump power threshold and 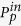 is the input pump power.

**Figure 8:**
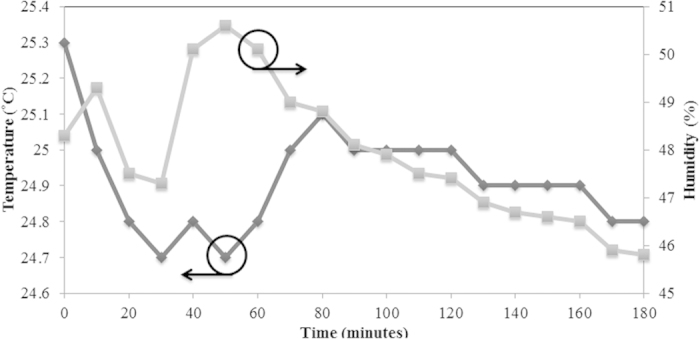
Work place temperature and humidity as a function of time over 180 minutes.

Figure 8 shows the changing of the temperature and humidity of the environment throughout the experiment, which was performed over a period of 180 minutes. Observation of Fig. 8 shows that the output power of the proposed setup remains relatively stable, and never exceeds a variation beyond 1 dB even with temperature and humidity fluctuations. Such evidence demonstrates that the system is largely stable and unaffected by external influences. That being said, external factors can influence the output power of the system, however in most cases the effect of the external influence is typically too minute to have noticeable consequences to the dual-wavelength output of the laser.

In this work, the PCF was used as the means to generate the dual-wavelength output. There have also been demonstrations of using saturable absorbers to generate a dual-wavelength output, such as graphene and graphene oxide. The approach of 24 uses a two-reflection peak fiber Bragg grating as the external cavity mirror together with a saturable absorber to create a Q-switched dual wavelength fiber laser, while the approach of 25 uses an arrayed waveguide grating to generate the desired tunable dual-wavelength output, which is then stabilized by a graphene based saturable absorber. Graphene oxide can also play the same role, as demonstrated in^26^, in which the graphene saturable absorber is used to stabilize the dual-wavelength output of a Nd:GYSGG laser. Besides this, there has also been a recent demonstration on the use of new materials as saturable absorbers, such as MoS_2_^27^ and black phosphorus^28^. In the case of 27 MoS_2_ is used in the form of a tapered fiber device to generate reasonably high-powered mode-locked pulses through evanescent field interactions. Currently, the interest of this lab would be to investigate MoS_2_ as a means of generating a stable dual-wavelength output using a PCF-based fiber laser. Most recently is the discovery of the use of black phosphorous as a saturable absorber, as has been discussed in detail in reference^28^. This finding will spur intense investigation into the development of black phosphorous as a viable alternative to graphene for saturable absorber applications.

## Conclusion

A stable dual wavelength laser operating at 1.9 μm and based on thulium-doped fiber as the gain medium has been proposed and demonstrated. The laser made use of the principle of polarization-dependent loss control to realize a dual-wavelength operation, and attained stability by utilization of a 10 cm long photonic crystal fiber. This photonic crystal fiber effectively acted as a Mach-Zehnder interferometer with a free spectral range of 0.2 nm. The dual wavelength thulium-doped fiber laser operated stably at room temperature in regards to both wavelength and power, and had a 45 dB optical signal-to-noise-ratio. The authors anticipate the success of the design described here will spur further research efforts and application in this area.

## Additional Information

**How to cite this article**: Soltanian, M. R. K. *et al.* A Stable Dual-wavelength Thulium-doped Fiber Laser at 1.9 µm Using Photonic Crystal Fiber. *Sci. Rep.* **5**, 14537; doi: 10.1038/srep14537 (2015).
